# The reproductive toxicity of aggregation-induced emission nanoparticles on mouse ovarian function

**DOI:** 10.1016/j.isci.2025.114312

**Published:** 2025-12-02

**Authors:** Yibin Zhang, Nan Qiao, Yihang Jiang, Miaozhuang Fan, Wenguang Zhang, Yue Jiao, Zhengzheng Li, Gang Feng, Wing-Cheung Law, Zhourui Xu, Gaixia Xu

**Affiliations:** 1 Guangdong Key Laboratory for Biomedical Measurements and Ultrasound Imaging, School of Biomedical Engineering, Shenzhen University Medical School, Shenzhen University, Shenzhen, Guangdong 518055, China; 2 Department of Industrial and Systems Engineering, Faculty of Engineering, The Hong Kong Polytechnic University, Hong Kong 999077, China

**Keywords:** Biological sciences, Nanoparticles, Optical imaging, Toxicology

## Abstract

Luminogens with aggregation-induced emission (AIEgens) have attracted increasing attention for biomedical applications, prompting concerns regarding their potential toxicity. Although some studies have reported the *in vivo* toxicity of AIEgens, their reproductive effects remain insufficiently characterized. In this study, we investigated the reproductive toxicity of a representative AIEgen, TPA-BT, encapsulated into nanoparticles (NPs), with a particular focus on ovarian function in mice. Oocyte maturation rates *in vitro*, body weight, and organ coefficients were evaluated following TPA-BT NPs exposure. Furthermore, oocyte apoptosis and serum hormone levels were examined. TPA-BT NPs significantly reduced oocyte maturation in a concentration-dependent manner. *In vivo*, high concentrations induced ovarian cell apoptosis, suppressed estrogen receptor (ERα and ERβ) expression, and disrupted anti-Müllerian hormone secretion, ultimately impairing reproductive function. These findings provide critical insights into the reproductive toxicity of AIEgens and establish a foundation for further mechanistic investigations to ensure their safe application in the biomedical field.

## Highlights

• The reproductive toxicity of AIEgens on mouse ovarian function was studied
• The study revealed that AIE NPs indirectly impact oocyte maturation via cumulus cells
• High TPA-BT NPs levels disrupt hormone expression and trigger ovarian cell apoptosis

## Graphical abstract

**Figure:**
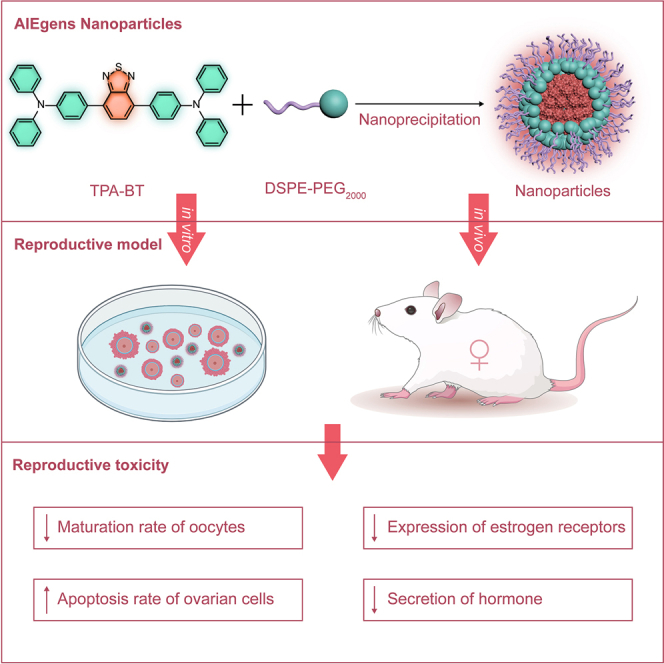

## Introduction

Organic fluorescent probes have emerged as indispensable agents in biomedical research, appreciated for their low safety risks and biodegradability.^1^^,^^2^^,^^3^^,^^4^^,^^5^^,^^6^^,^^7^ Nevertheless, they continued to suffer from poor water solubility, severe photobleaching, and limited stability, significantly limiting their broader application in the field. In recent years, luminogens with aggregation-induced emission characteristics (AIEgens) have gained widespread use in various biomedical applications, including vascular imaging, tumor imaging, image-guided surgery, photothermal therapy, and photodynamic therapy, owing to their remarkable colloidal stability in water, good resistance to photobleaching, and significantly enhanced functional stability.^8^^,^^9^^,^^10^^,^^11^^,^^12^^,^^13^^,^^14^^,^^15^ These attributes positioned AIEgens as a superior alternative to traditional organic probes.^16^^,^^17^^,^^18^^,^^19^^,^^20^ However, their clinical application and broader use are heavily constrained by concerns regarding their toxicity.^21^ Consequently, the potential toxicity of AIEgens to biological systems remains a critical issue that warrants thorough investigation in the years ahead. While most existing research on the toxicity of AIEgens has focused on cytotoxicity and toxicity to major organs, studies addressing their reproductive toxicity were scarce.^20^^,^^21^^,^^22^^,^^23^^,^^24^ Given that reproductive toxicity is a critical factor in the development and clinical application of AIEgens and considering that the reproductive system is the foundation for species continuity and diversity, as well as a vital component in the evolution and development of species, it is, therefore imperative to conduct a systematic and detailed assessment of the reproductive toxicity of AIEgens before their application in biomedical fields.

In this study, we selected, synthesized, and characterized a classic AIEgens, 4,4′- (benzo[c][1,2,5]thiadiazole-4,7-diyl)bis(N, N-diphenylamine) (TPA-BT), to evaluate its reproductive toxicity.^25^^,^^26^^,^^27^^,^^28^^,^^29^^,^^30^^,^^31^^,^^32^ Initially, immature oocytes were co-cultured with TPA-BT NPs *in vitro* to assess NPs uptake and oocyte development. The results showed that although TPA-BT NPs could be taken up by the cumulus cells surrounding the oocyte, they could not enter the oocytes. Notably, the presence of TPA-BT NPs significantly reduced the oocyte maturation rate. Subsequently, six-week-old female Kunming mice were administered varying concentrations of TPA-BT NPs to investigate effects on blood, major organs, ovarian, and hormones. High concentrations of TPA-BT NPs (>20 mg/kg) were found to induce substantial ovarian cell apoptosis, decrease the expression of estrogen receptors ERα and ERβ, and disrupt the secretion of anti-Müllerian hormone (AMH), potentially leading to reproductive dysfunction. These findings offered important insights into the biosafety profile of AIEgens and provided valuable guidance for developing safer AIEgens tailored for specific biomedical applications.

## Results and discussion

### Optical properties of the triphenylamine-benzo[c][1,2,5]thiadiazole

**Figure 1:**
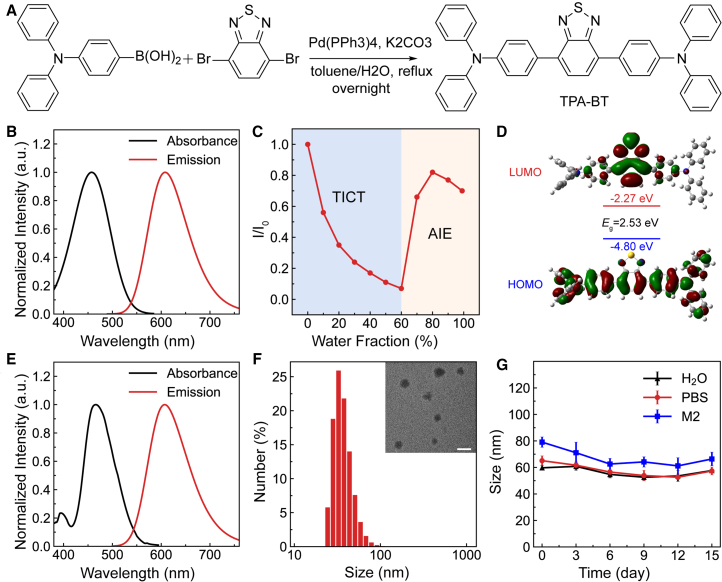
Optical properties of TPA-BT and TPA-BT NPs (A) General synthetic procedure for the preparation of TPA-BT. (B) Normalized absorption and fluorescence spectra of AIEgens in THF. The excitation wavelength is 458 nm, and the concentration is 10 μM. (C) Relative emission intensity (*I*/*I*_0_) of AIEgcens versus water fraction. (D) Chemical structures and optimized ground state (S_0_) geometries of TPA-BT. The excitation wavelength is 460 nm, and the concentration is 10 μM. (E) Normalized absorption and fluorescence spectra of NPs in aqueous solutions. (F) DLS profile of TPA-BT NPs. Inset: TEM image of TPA-BT NPs (scale bars, 100 nm). (G) Hydrodynamic size of TPA-BT NPs under different conditions over 14 days. Error bars represent the mean ± SD of three independent experiments.

The synthetic route of TPA-BT is shown in Figure 1A. The optical properties of TPA-BT were investigated using ultraviolet-visible (UV-vis) and fluorescence spectrometers. The absorption and emission peaks of TPA-BT in Tetrahydrofuran (THF) were observed at 458 nm and 608 nm, respectively (Figures 1B and S8). Additionally, its aggregation-induced emission (AIE) characteristics were studied in a THF/H_2_O solvent system (Figure 1C). As the water fraction increased, the fluorescence emission intensity of TPA-BT exhibited a gradual decrease, which can be ascribed to the enhanced twisted intramolecular charge transfer (TICT) effect.^33^ With a further increase in water content, however, a pronounced enhancement of the fluorescence intensity was observed for the AIEgens, primarily resulting from the restriction of intramolecular motions along with molecular aggregation (Figure S7).^16^ The optical properties of TPA-BT were analyzed through density functional theory (DFT) calculations. As shown in Figure 1D and Table S1, the electron cloud of the highest occupied molecular orbital (HOMO) was localized on the triphenylamine (TPA) unit, while the lowest unoccupied molecular orbital (LUMO) was situated on the benzo[c][1,2,5]thiadiazole (BT) unit, with a calculated band gap value of 2.53 eV (Figure S6). These findings indicated that TPA-BT exhibited a distinct intramolecular charge transfer (ICT) characteristic, which led to gradually reducing fluorescence intensities in the solvent with increasing polarity, e.g., the mixture solvent with less than 60% water mentioned above. The dihedral angles within TPA-BT were also calculated, with the angles between adjacent aromatic rings in TPA and between TPA and BT being 69.03°, 68.62°, and 34.24°, respectively (Figure S5). This torsional molecular backbone effectively prevents π-π stacking, thereby enabling a prominent AIE of TPA-BT upon aggregation and a highly boosted fluorescence emission as the water fraction is larger than 60%. Remarkably, an exceptionally high quantum yield of 55% of the solid-state TPA-BT has been determined, thus making it suitable to observe and study the subsequent reproductive toxicity analysis.

To facilitate the use of AIEgens in biomedical applications, TPA-BT was formulated into nanoparticles (NPs) using DSPE-PEG_2000_ as the capping agent via a nanoprecipitation method, and its morphology and photophysical properties were thoroughly investigated. As shown in Figure 1E, the absorption and emission peaks of TPA-BT NPs in water were observed at 460 nm and 610 nm, respectively. Dynamic light scattering (DLS) measurements revealed that the hydrodynamic size of TPA-BT NPs was 59.7 ± 1.5 nm, with a zeta potential of −2.64 mV (Figure 1F; Table S5). Transmission electron microscopy (TEM) images indicated that the average size of the TPA-BT NPs was approximately 40 nm. Importantly, even after two weeks of storage under ambient conditions, both the fluorescence intensity (Figures S10 and S11) and hydrodynamic size (Figure 1G) of TPA-BT NPs remained stable in phosphate-buffered saline (PBS) and M2 medium, demonstrating excellent colloidal stability.

### Effect of triphenylamine-benzo[c][1,2,5]thiadiazole nanoparticles on oocyte maturation *in vitro*

**Figure 2:**
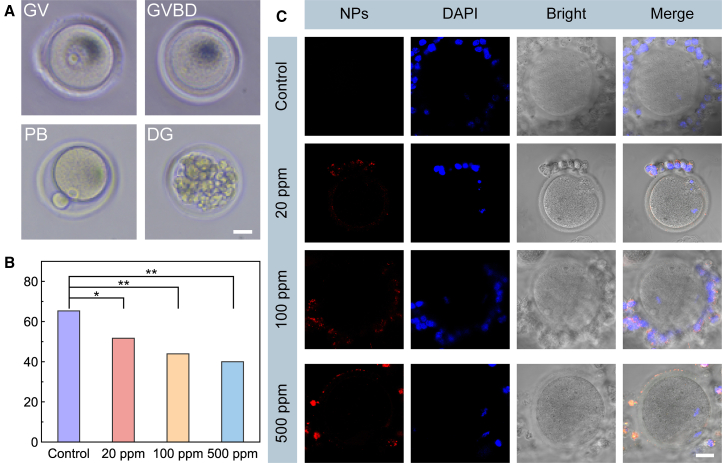
The impact of TPA-BT NPs on the maturation rate of oocytes cultured *in vitro* (A) Oocytes at different stages of meiosis. The oocytes at the GV stage are in the prophase of meiosis, characterized by a large nucleus, highly dispersed chromatin, and an intact nuclear membrane, known as the germinal vesicle. At the GVBD stage, the oocytes have initiated meiosis but have not yet entered metaphase of the second meiotic division, and the germinal vesicle has ruptured. Oocytes at the PB stage have entered metaphase of the second meiotic division, also known as the maturation stage. DG may occur at any stage due to intrinsic or extrinsic factors. (Scale bars, 20 μm). (B) Proportions of oocytes at various stages of meiosis after 18 h of *in vitro* culture with varying concentrations of TPA-BT NPs. (c) CLSM images of oocytes after 18 h of *in vitro* culture with varying concentrations of TPA-BT NPs. (Scale bars, 10 μm). *p* values were calculated using the chi-square test, ∗*p* < 0.05; ∗∗*p* < 0.01.

As illustrated in Figure 2A, oocyte meiosis was divided into several stages based on morphological classification. The germinal vesicle (GV) phase corresponds to the prophase of meiosis, characterized by a prominent nucleus, highly dispersed chromatin, and an intact nuclear membrane.^34^ The germinal vesicle breakdown (GVBD) phase denotes the onset of meiosis, during which the germinal vesicle ruptures but the oocyte has not yet reached metaphase II. The polar body (PB) stage indicates progression to metaphase II, also referred to as the maturation phase. In addition, an oocyte may undergo degeneration (DG) at any stage of meiosis, resulting from intrinsic factors such as DNA abnormalities and mitochondrial dysfunction, or from extrinsic influences including oxidative stress, in concert with natural apoptotic pathways.^35^^,^^36^^,^^37^

To investigate the impact of TPA-BT NPs on oocyte maturation, we co-cultured cumulus-oocyte complexes (COCs) with varying concentrations of TPA-BT NPs for 18 h (Figure S9). After removing the surrounding cumulus cells, we assessed the growth and development of the oocytes using an aberration-corrected microscope and recorded the number of oocytes that remained arrested at different stages of meiosis. As shown in Figure 2B and Table S2, TPA-BT NPs influenced the meiotic process, resulting in an increase in the number of oocytes arrested at the GV and GVBD stages, thereby reducing the number reaching the PB stage and ultimately lowering the oocyte maturation rate. Statistical analysis revealed significant differences in the number of oocytes in the PB stage across different groups (*p* < 0.05). Additionally, TPA-BT NPs may lead to oocyte inactivity, further contributing to degeneration and apoptosis, as indicated by an increase in the number of oocytes at the DG stage. These findings suggested that TPA-BT NPs can, to some extent, disrupt the meiotic process, inhibit the maturation of oocytes *in vitro*, and promote oocyte degeneration.

To explore the mechanisms by which TPA-BT NPs affect oocyte maturation, COCs were co-cultured with TPA-BT NPs for 18 h, followed by a 4-h co-culture with 4′,6-diamidino-2-phenylindole (DAPI), and then imaged using a confocal laser scanning microscope (CLSM). As illustrated in Figures 2C and 2A significant accumulation of TPA-BT NPs was observed exclusively in the cumulus cells surrounding the oocytes, with strong colocalization with the DAPI probe. This finding suggested that the cumulus cells encircling the oocytes provided substantial protection, effectively preventing TPA-BT NPs from entering the oocytes. Additionally, after immunofluorescence staining of GVBD and PB stage oocytes co-cultured with TPA-BT NPs, CLSM imaging was performed to examine spindle morphology. As shown in Figure S12, the spindle structures in oocytes from all groups appeared normal, with chromosomes aligned properly on the equatorial plate.^38^ These results confirmed that the influence of TPA-BT NPs on oocyte maturation is indirect, primarily mediated through their effects on the surrounding cumulus cells.^39^^,^^40^

**Figure 3:**
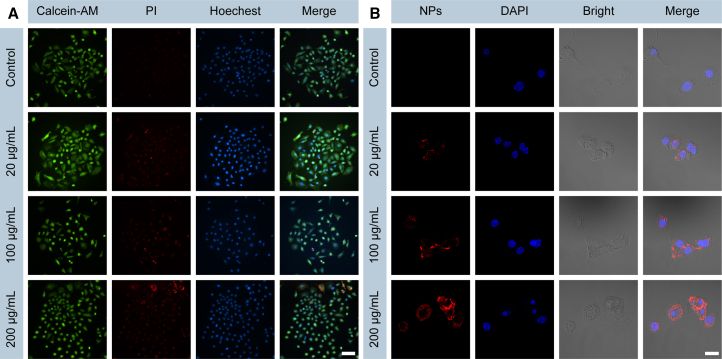
Toxicology evaluation of cumulus cells exposed to TPA-BT NPs (A) CLSM images of live/dead staining of cumulus cells after 18 h of *in vitro* culture with varying concentrations of TPA-BT NPs. (Scale bars, 100 μm). (B) CLSM images of cumulus cells after 18 h of *in vitro* culture with varying concentrations of TPA-BT NPs. (Scale bars, 10 μm).

To explore the underlying cause of cumulus cell dysfunction induced by TPA-BT NPs, we initially examined the cytotoxicity of TPA-BT NPs on cumulus cells. Cumulus cells were co-cultured with varying concentrations of TPA-BT NPs for 18 h, after which cell viability was assessed. As shown in Figure S13, even at a high concentration of 500 μg/mL, a cell survival rate of 82% was observed, indicating that TPA-BT NPs exhibited low toxicity toward cumulus cells. Additionally, live/dead staining was performed on cumulus cells co-cultured with TPA-BT NPs. As illustrated in Figure 3A, no significant apoptosis was observed even at a high concentration of TPA-BT NPs (200 μg/mL), further confirming the minimal toxicity of TPA-BT NPs on cumulus cells. Moreover, we investigated the uptake of TPA-BT NPs by cumulus cells. As shown in Figure 3B, the uptake of NPs by cumulus cells increased with higher concentrations of TPA-BT NPs. In summary, we proposed that the uptake of TPA-BT NPs by cumulus cells disrupted their steroidogenesis and hormone production, leading to functional impairments that subsequently affected oocyte development and maturation.^41^

### Histopathologic changes in the ovaries and major organs

**Figure 4:**
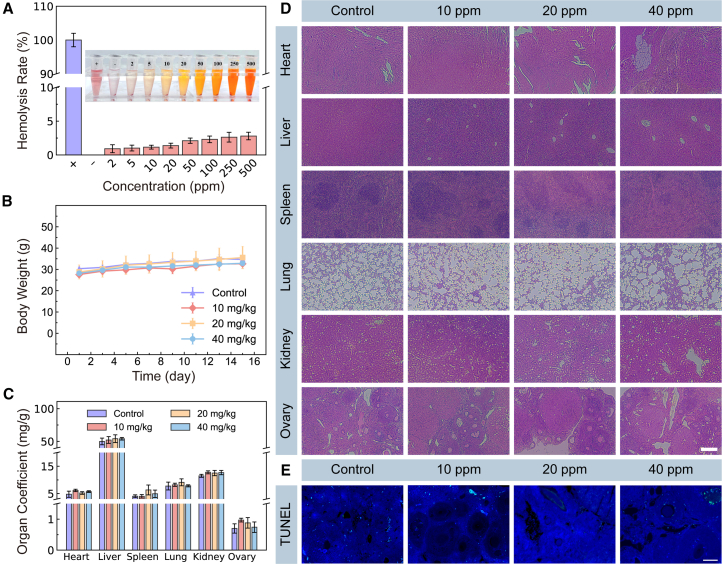
Organ toxicology evaluation of TPA-BT NPs (A) Hemolysis rate of TPA-BT NPs at different concentrations. (B) Body weight of female Kunming mice treated with various concentrations of TPA-BT NPs. (C) Organ coefficients of Kunming mice treated with various concentrations of TPA-BT NPs. (D) Histological images of the ovaries and major organs of Kunming mice treated with various concentrations of TPA-BT NPs. (Scale bars, 200 μm). (E) TUNEL staining images of ovarian apoptosis in Kunming mice injected with different concentrations of TPA-BT NPs. (Scale bars, 30 μm). Error bars represent the mean ± SD of independent experiments.

A hemolysis assay was conducted to evaluate the biocompatibility of TPA-BT NPs. As shown in Figure 4A, even at high concentrations (500 ppm), the hemolysis rate remained low (less than 3.5%), attributed to DSPE-PEG_2000_ as a capping agent, effectively preventing TPA-BT NPs from leaking. Further toxicity assessments were carried out by injecting different concentrations of TPA-BT NPs into mice. As illustrated in Figure 4B, mice’s body weights in all groups steadily increased over 15 days, indicating minimal systemic side effects. To further investigate the immunogenicity and toxicity of TPA-BT NPs, a blood routine test (Table S3) and a blood biochemistry test (Table S4) were conducted. The results showed no significant changes in 17 hematological and 8 organ function indicators compared to the PBS control group, indicating that TPA-BT NPs exhibited no acute toxicity. Major organs, including the heart, liver, spleen, lungs, and kidneys, were collected to assess organ coefficients, and given the focus on reproductive toxicity, the ovarian organ index was also recorded. As shown in Figure 4C, no abnormal growth was observed in the major organs or ovaries. Further histological analysis of tissue sections from both control and experimental groups was performed. As depicted in Figure 4D, no tissue necrosis or lymphocyte infiltration was observed in histological samples. The cellular morphology in tissue sections from the experimental group mice appeared normal, closely resembling that of the control group. Moreover, no fibrosis, glomerular, or tubular abnormalities were detected in the lung, liver, or kidney sections, and no abnormalities were found in the ovarian sections. These findings indicated that TPA-BT NPs exhibited good biocompatibility, low safety risks, and low reproductive toxicity at the tested concentrations.

Next, TUNEL assays were performed to assess apoptosis in ovarian cells. As shown in Figure 4E, apoptotic cells were labeled with fluorescein isothiocyanate (FITC), appearing green, while the nuclei of normal ovarian cells were stained blue. The percentage of the area occupied by apoptotic cells in the stained sections was calculated, with the results presented in Figure S14. A significant difference (*p* < 0.05) in the area of ovarian cell apoptosis was observed in the high-concentration experimental group (>20 mg/kg) compared to the control group. Western blot (WB) analysis was carried out to further confirm the obtained results (Figure S15). These findings suggested that low-dose injections of TPA-BT NPs (<20 mg/kg) did not result in significant ovarian cell apoptosis, whereas high-dose injections of TPA-BT NPs (>20 mg/kg) led to pronounced ovarian cell apoptosis, potentially impairing reproductive function in female mice.

### Changes in estrogen receptor expression and hormone levels

**Figure 5:**
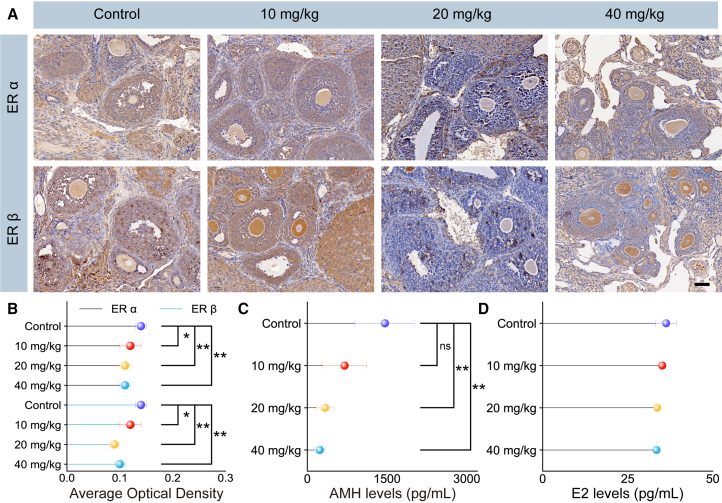
Reproductive toxicology evaluation of TPA-BT NPs (A) Immunohistochemical staining of ERα and ERβ in ovarian sections of mice injected with varying concentrations of TPA-BT NPs. (Scale bars, 50 μm). (B) Statistical analysis of the mean optical density of ERα and ERβ positive signals. (C) Detection of anti-Müllerian hormone levels in mouse serum 14 days after TPA-BT NPs injection. (D) Detection of estradiol hormone level in mouse serum 14 days after TPA-BT NPs injection. Error bars represent the mean ± SD of independent experiments. *p* values were calculated using Student’s *t* test, ∗*p* < 0.05; ∗∗*p* < 0.01.

Estrogen receptors play a crucial role in maintaining the differentiation of ovarian cumulus cells and are essential for the growth, development, and ovulation functions of follicles and oocytes. Dysfunctions in estrogen and its receptors are closely associated with clinical reproductive endocrine disorders such as polycystic ovary syndrome (PCOS) and endometriosis (EMS).^42^^,^^43^ Therefore, evaluating estrogen receptors is vital for understanding the reproductive toxicity of TPA-BT NPs in mice. Estrogen receptor alpha (ERα) and estrogen receptor beta (ERβ) are critical mediators of estrogen’s biological effects, encoded by the genes *ESR1* and *ESR2*, which are located on non-homologous chromosomes, respectively.^42^^,^^44^ Furthermore, the expression of ERα and ERβ varies significantly across different tissues and cells. ERα is primarily expressed in the uterus, ovaries, and mammary glands, while ERβ is mainly found in the nervous, ovarian, and cardiovascular systems.^45^ In this study, immunohistochemical staining was used to detect ERα and ERβ in ovarian sections, as shown in Figure 5A. Brownish-yellow staining indicates the expression of ERα and ERβ, while blue represents nuclear staining. The results demonstrated that as the concentration of TPA-BT NPs increased, the expression levels of ERα and ERβ in the ovarian sections of mice gradually decreased. The average optical density of the immunohistochemical staining results was quantified using ImageJ software. As illustrated in Figure 5B, the expression of ERα and ERβ in the ovarian sections of female mice decreased significantly compared to the control group (*p* < 0.05), regardless of whether low concentrations (10 mg/kg) or high concentrations (>20 mg/kg) of TPA-BT NPs were administered. These findings suggested that TPA-BT NPs exhibited substantial reproductive toxicity by reducing the expression of estrogen receptors in the ovaries of female mice, thereby impairing their reproductive capacity.

Anti-Müllerian hormone (AMH) is a secreted glycoprotein hormone that plays a crucial role in reproductive development and regulation.^46^ In males, AMH ensures the regression of the Müllerian ducts, and loss of AMH function can result in the persistence of Müllerian duct derivatives, such as the uterus and fallopian tubes.^47^^,^^48^^,^^49^ In females, AMH acts as a negative regulator of folliculogenesis, and disruptions in its signaling pathway can interfere with or inhibit normal follicular development and function, potentially leading to conditions such as PCOS and Primary Ovarian Insufficiency (POI).^50^ Estradiol, a form of estrogen primarily secreted by the ovaries, is essential for developing and maintaining the female reproductive system.^51^ Therefore, measuring the levels of AMH is important in studying the reproductive toxicity of TPA-BT NPs in mice. After injecting mice with different concentrations of TPA-BT NPs and continuously feeding them for 14 days, fresh blood was collected for serum AMH and estradiol level analysis, followed by statistical evaluation. As shown in Figure 5C, there was no significant difference in serum AMH levels between the control group and the mice injected with a low dose (10 mg/kg) of NPs. However, a significant difference (*p* < 0.01) was observed in the serum AMH levels of mice injected with high doses (>20 mg/kg) of NPs compared to the control group. And the effects of TPA-BT NPs on preantral follicles in mice were statistically analyzed to provide further evidence (Figure S16 and Table S6). In contrast, estradiol levels did not show any significant differences across the experimental groups and the control group (Figure 5D). These findings suggested that high doses of TPA-BT NPs reduced AMH levels in mice, which may adversely affect the reproductive function of female mice, while estradiol levels remain unaffected. Typically, the concentration of TPA-BT NPs used in mice for biomedical applications ranges from 5 to 15 mg/kg, with no evident reproductive toxicity observed at these levels. However, for the future clinical applications of AIEgens-based NPs, more comprehensive toxicity assessments are crucial to ensure their complete safety and absence of any side effects on biological systems.^25^^,^^27^

This study is the first to report the reproductive toxicity of AIEgens-based NPs on mouse ovarian function. TPA-BT NPs were observed to cause dysfunction in the cumulus cells, indirectly disrupting oocyte maturation. Histopathological examination revealed no adverse effects of TPA-BT NPs on the structure of the ovaries or major organs in female mice. However, TUNEL staining indicated that high concentrations of TPA-BT NPs (>20 mg/kg) induced significant ovarian cell apoptosis. Additionally, immunohistochemical staining confirmed that, fourteen days after a single intravenous injection, high concentrations of TPA-BT NPs (>20 mg/kg) led to the reduced expression of estrogen receptors ERα and ERβ, as well as anti-Müllerian hormone (AMH). These findings suggested that high concentrations of TPA-BT NPs (>20 mg/kg) exhibited significant reproductive toxicity in mice, ultimately impairing their reproductive function. Given the tremendous potential of AIEgens in biomedical applications, we hope this research will provide valuable insights into the design and synthesis of safer AIEgens, thereby greatly enhancing their prospects for future clinical application.

### Limitations of the study

Although the study comprehensively evaluated the effects of a single intravenous injection of TPA-BT nanoparticles (NPs) on the reproductive system after 14 days, it failed to capture potential chronic or long-term reproductive toxicity. In future studies, we plan to implement a repeated dosing regimen and extend the observation period (e.g., several months) to assess chronic toxicity. This may include monitoring reproductive outcomes, such as offspring health, to provide a more comprehensive toxicity profile.

## Resource availability

### Lead contact

The relevant experimental reagents, experimental methods, and related data of this study can be obtained by contacting Gaixia Xu (xugaixia@szu.edu.cn).

### Materials availability

This study did not generate new unique reagents.

### Data and code availability

• All data reported in this article will be shared by the lead contact upon request.
• This article does not report the original code.
• Any additional information required to reanalyze the data reported in this article is available from the lead contact upon request.

## Acknowledgments

The authors acknowledge the financial support from the 10.13039/501100001809National Natural Science Foundation of China (62205216), the Basic Research Foundation of Shenzhen (JCYJ20220531101814034, JCYJ20230808105412024), the Medical-Engineering Interdisciplinary Research Foundation of Shenzhen University (2023YG002, 2024YG009), the Scientific Instrument Developing Project of Shenzhen University (2023YQ008), and the Start-up Grant from 10.13039/501100009019Shenzhen University (868-000001032015). The authors also thank the Instrument Analysis Center of Shenzhen University for the equipment used. All animal operations complied with the regulations of the Animal Ethics and Welfare Committee of Shenzhen University.

## Author contributions

Y.Z. collected the data and wrote the article. Y.Z. and N.Q. designed the biological experiments and conducted the *in vitro* oocyte experiments. Y. J. and M. F. analyzed the results. W. Z. and Y. J. conducted the *in vivo* animal experiments. Z. L. and G. F. synthesized the AIEgens and NPs. W. L. and Z. X. reviewed and edited the article. G. X. supervised the entire project.

## Declaration of interests

The authors declare that they have no competing interests.

## STAR★Methods

### Key resources table

**Table:** 

REAGENT or RESOURCE	SOURCE	IDENTIFIER
**Chemicals, peptides, and recombinant proteins**		

Distearoyl-sn-glycero-3-phosphoethanolamine-N-(methoxy-(polyethylene glycol)-2000)	Xi’an ruixi Biological Technology	LP-R4-039; CAS:147867-65-0
Calcein-AM	Thermo Fisher Scientific	C3100MP; CAS: 148504-34-1
Propidium Iodide	Thermo Fisher Scientific	P1304MP; CAS: 25535-16-4
2'-(4-Ethoxyphenyl)-5-(4-methylpiperazin-1-yl)-1H,1′H-2,5′-bibenzo[d]imidazole	Sigma-Aldrich	382065; CAS: 23491-52-3
4-(Diphenylamino)phenylboronic acid	Sigma-Aldrich	647292; CAS: 201802-67-7
4,7-Dibromobenzo[c]-1,2,5-thiadiazole	Sigma-Aldrich	693847; CAS: 15155-41-6
Pd(dppf)Cl_2_	Sigma-Aldrich	697230; CAS: 72287-26-4
ACTIN	Servicebio	GB15003
BCL-2	Servicebio	GB154380
BAX	Servicebio	GB15690
Caspase3	Servicebio	GB11767C

**Critical commercial assays**		

Cell counting kit-8	Beyotime Biotechnology	C0038

**Experimental models: Organisms/strains**		

Kunming mice	GUANGDONG MEDICAL LABORATORY ANIMAL CENTER	SPF level Kunming female mice

**Software and algorithms**		

ImageJ	Open source	N/A
GraphPad Prism	GraphPad	https://www.graphpad.com/

### Method details

#### Materials and characterization

The aggregation-induced emission luminogen 4,4′-(benzo[c][1,2,5]thiadiazole-4,7-diyl)bis- (N, N-diphenylamine) (TPA-BT) was synthesized by us, and the synthesis details are described below. Chloroform and ethanol were obtained from the Macklin reagent. 2- Distearoyl-sn-glycero-3-phosphoethanolamine-N-[methoxy- (polyethylene glycol)-2000 (DSPE-mPEG_2000_) was purchased from Xi’an Ruixi Biological Technology Co., Ltd. PBS (pH 7.4) was purchased from Beyotime Biotechnology. The Cell Counting Kit-8 (CCK-8) was purchased from Hangzhou Fude Biological Technology Co., Ltd. M2 medium was purchased from Sigma-Aldrich. Dulbecco’s Modified Eagle’s Medium (DMEM), fetal bovine serum (FBS), penicillin, and streptomycin were all obtained from Gibco.

^1^H and ^13^C NMR spectra were recorded using a Bruker ARX 400 spectrometer using CDCl_3_ or CD_2_Cl_2_ as the solvent. High-resolution mass spectra (HRMS) were recorded using a GCT premier CAB048 mass spectrometer operated in MALDI-TOF mode. The extinction spectra were measured using a UV−vis scanning spectrophotometer (4100, Agilent, USA). Steady-state fluorescence spectra were obtained on a spectrofluorometer (F4600, Hitachi, Japan). The hydrodynamic diameter of TPA-BT NPs was determined by dynamic light scattering on a particle size analyzer (Zetasizer Nano ZS90, Malvern Panalytical, Malvern, United Kingdom) with a 90° dangle. The morphologies of TPA-BT NPs were recorded using TEM (HT-7700, Hitachi, Japan) operating at 160 kV in bright-field mode. The chemical structures of the final products have been confirmed by NMR spectra and mass spectra (Figures S1–S4).

#### Animal handling

The protocol for the animal experiments was approved by the Animal Ethical and Welfare Committee of Shenzhen University (AEWC-SZU, AEWC-202300019). The experiments were conducted strictly with governmental and international guidelines on animal experimentation. 4–6 weeks old female Kunming mice were purchased from the Guangdong Medical Laboratory Animal Center. Kunming mice were used with average weights of 30 g. According to Biosafety and Animal Ethics requirements, all efforts were made to minimize the number of animals used and reduce their suffering.

#### Synthesis of TPA-BT

The compound 4,4′- (benzo[c][1,2,5]thiadiazole-4,7-diyl)bis(N, N-diphenylamine) (TPA- BT) was synthesized according to previous report.^52^ A solution of 4-(Diphenylamino)phenylboronic Acid (112 mg, 0.18 mmol) in ethanol (3 mL), together with aqueous 2 M Na_2_CO_3_ (10 mL), was introduced under an argon atmosphere at 60 °C into a benzene suspension (20 mL) containing 4,7-Dibromo-2,1,3-benzothiadiazole (46 mg, 0.16 mmol) and tetrakis(triphenylphosphine)palladium(0) (5.5 mg, 0.0048 mmol). The resulting mixture was heated at 85 °C for 12 h. Upon completion, the reaction was quenched with water (50 mL) and extracted three times with toluene (30 mL each). The combined organic fractions were dried over anhydrous MgSO_4_ and concentrated under reduced pressure. Purification of the crude product by silica gel chromatography (KANTO 60N; chloroform/hexane = 1:2) afforded an orange solid (50 mg), consisting of a mixture of TPA-BT and 4-bromo-7-[4-(N,N-diphenylamino)phenyl]-2,1,3-benzothiadiazole.

This orange material (50 mg) was subjected to a second coupling step. A mixture of the crude solid and tetrakis(triphenylphosphine)palladium(0) (3.8 mg, 0.0033 mmol) in benzene (15 mL) was treated under argon at 60 °C with a solution of 4-(Diphenylamino)phenylboronic Acid (124 mg, 0.21 mmol) in ethanol (1 mL), followed by aqueous 2 M Na_2_CO_3_ (7 mL). The suspension was then heated at 85 °C for 12 h. After cooling, the reaction mixture was poured into water (50 mL) and extracted with toluene (3 × 20 mL). The combined organic layers were dried over anhydrous MgSO_4_ and concentrated in vacuo. Subsequent purification by silica gel column chromatography (KANTO 60N; chloroform/hexane = 1:2) delivered TPA-BT as a red powder (52 mg, 0.084 mmol, 53%). An analytically pure sample was obtained by gel-permeation chromatography using chloroform as the eluent.

Spectral data of TPA-BT.^53^ ^1^H NMR (600 MHz, CDCl_3_) δ 7.90−7.86 (m, 4H), 7.75 (s, 2H), 7.32−7.27 (m, 8H), 7.24−7.17 (m, 12H), 7.07 (tt, J = 7.3, 1.2 Hz, 4H). ^13^C NMR (151 MHz, CDCl_3_) δ 154.1, 148.0, 147.5, 132.2, 131.0, 129.9, 129.3, 127.4, 124.9, 123.3, 122.9.

#### Fabrication of AIEgens NPS

The fabrication of AIEgens NPs was performed by injecting a THF solution (0.5 mL) containing AIEgens (1 mg) and DSPE-PEG_2000_ (5 mg) into 5 mL of ultrapure water under vigorous stirring for 2 minutes. The resulting NPs were then purified by dialysis (molecular weight cutoff of 100 kDa) against ultrapure water for 24 hours. Subsequently, the AIEgens NPs were concentrated using an ultrafiltration tube (molecular weight cutoff of 100 kDa) at 4400 rpm for 20 minutes. The collected AIEgens NPs were redispersed in 1 × PBS buffer (pH 7.4) and stored at 4 °C dark.

#### Measurement of absolute η

The absolute η was determined using the C1137 system, which consists of a monochromatic light source (150 W xenon lamp), an integrating sphere, a photonic multichannel analyzer, and η Measurement Software U6039–05. The parameter η is defined as the ratio of the number of photons emitted by a sample to the number of photons absorbed by the sample. For the water dispersion of TPA-BT NPs (10 μM), excitation light at 460 nm was used. Blank NPs, composed of DSPE-PEG2000 with the same size range as the AIEgen NPs, served as the blank, and a quartz cuvette was used as the sample holder. The fluorescence spectra of the AIEgen NPs were recorded under the respective excitation conditions. The η values were then automatically calculated using the Measurement Software U6039–05. After each measurement, the quartz cuvette was cleaned with dichloromethane and ethanol to prevent contamination of the integrating sphere and minimize measurement errors for subsequent tests. The measured quantum yield (QY) of the AIEgen NPs was found to be 45.5%, with a standard deviation of 0.0577.

#### Collection and *in vitro* culture of oocyte

Firstly, prepare the pregnant mare serum gonadotropin (PMSG) by adding 10 mL of diluent to a 1000 IU vial of the solid powder using a disposable syringe. Gently shake the vial to dissolve and mix the solution thoroughly, then set it aside for later use. An intraperitoneal injection of 10 IU PMSG was administered to the experimental mice to induce ovulation. After 48 hours, the oocyte retrieval procedure begins. Prepare the oocyte handling and culture media a few hours before the experiment. Dispense the culture media onto culture dishes and cover them with mineral oil. Transfer the culture dishes containing the media and handling solutions into a CO_2_ incubator for 2 hours for proper incubation. The oocyte culture medium consisted of M2 medium supplemented with fetal bovine serum, penicillin–streptomycin, human chorionic gonadotropin (hCG), and follicle-stimulating hormone (FSH).

At the start of the experiment, euthanize the mice using cervical dislocation and promptly extract both ovaries, placing them into a well containing the handling solution. In a laminar flow hood, use a disposable syringe needle to mince the ovaries and collect the immature oocytes using a self-made oocyte retrieval needle. Transfer the oocytes to a culture dish containing droplets of the culture medium, with approximately 10 oocytes in each droplet. Next, place the culture dish containing the oocytes into a CO_2_ incubator (37 °C, 5% CO_2_, saturated humidity) for 18 hours of culture. It is crucial that the entire process, from the euthanasia of the mouse to the placement of the oocyte culture dish in the incubator, be completed within 30 minutes. This time constraint is essential because oocytes are sensitive to light, and prolonged exposure under a microscope may negatively impact their subsequent growth and development.

#### Collection and *in vitro* culture of cumulus cells

Similar to the method described above for oocyte retrieval, begin by administering an intraperitoneal injection of PMSG to the experimental mice. After 48 hours, euthanize the mice and promptly extract the bilateral ovaries, placing them in sterile PBS to cleanse the surrounding fatty tissue. After thoroughly washing the ovaries, transfer them to DMEM culture medium containing 10% FBS and 1% penicillin-streptomycin. Within a laminar flow hood, proceed to isolate the ovarian cumulus cells mechanically. Place the dish containing the ovaries under an inverted optical microscope and use a 1 mL disposable syringe needle to puncture the mature follicles, allowing the follicular fluid containing oocytes and cumulus cells to flow into the DMEM culture medium. Filter the DMEM culture medium containing the ovarian tissue and follicular fluid through a cell strainer. Centrifuge the filtered liquid at 1200 rpm for 5 minutes, discard the supernatant, and add 5 mL of DMEM culture medium. Gently pipette the solution a few times to create a cumulus cell suspension, then transfer it into a T25 culture flask and place it in a CO_2_ incubator (37 °C, 5% CO_2_, saturated humidity) for culture. Replace the culture medium every 48 hours and regularly observe the cells under an inverted microscope, capturing images to monitor cell health and growth.

#### Effects of TPA-BT NPs on oocyte morphology and maturation rate

After co-culturing oocytes with NPs *in vitro* for 18 hours, transfer them to a hyaluronidase solution. The hyaluronidase should be pre-diluted to a 0.1% concentration with PBS and heated to 37 °C in a water bath. Once the cumulus cells on the exterior of the oocytes have detached, observe the morphological changes of the oocytes under an inverted microscope. Determine the stage of the cells—whether they are in the germinal vesicle (GV) phase, germinal vesicle breakdown (GVBD) phase, polar body (PB) phase, or degradation (DG) phase. Count the number of oocytes in each stage and photograph their morphological structures. Finally, the *in vitro* maturation rate of the oocytes (maturation rate = number of PB stage oocytes/total number of oocytes) will be calculated based on the statistical results, and statistical analysis will be performed on the obtained data.

#### Effects of TPA-BT NPs on oocyte invasiveness

Select cumulus-oocyte complexes (COCs) with fewer cumulus cells or denuded oocytes (without cumulus cells) from four groups of culture media and wash them three times in PBS. After washing, transfer the cells to 4% paraformaldehyde (PFA) for 20 minutes to fix them. During the transfer process, ensure each cell is as dispersed as possible. After fixation, transfer the cells to a DAPI staining solution for 20 minutes. Finally, images of the cells were captured using the confocal laser scanning microscope (CLSM) ZEISS LSM880 to observe and document their morphology and staining patterns.

#### Effects of TPA-BT NPs on oocyte spindle

Immunofluorescence staining is required to label microtubules and chromosomes in oocytes to capture the structural features of spindles and chromosomes using the CLSM. The reagents needed for the immunofluorescence staining experiment include PBS, PBST (wash buffer), 4% paraformaldehyde (PFA), 0.5% Triton X-100 (permeabilization buffer), 5% bovine serum albumin (BSA) (blocking solution), Alpha Tubulin antibody (primary antibody for microtubules), Alexa Fluor 488 (secondary antibody for microtubules), and DAPI. The steps for the immunofluorescence staining experiment include fixation, permeabilization, blocking, primary antibody incubation, secondary antibody incubation, and DAPI staining.

Fixation: Wash the oocytes with PBS, select oocytes with well-preserved morphology in the GVBD and PB stages, and use an oocyte retrieval needle to transfer the cells to a well containing 4% PFA. Fix the cells at room temperature in the dark for 30 minutes. After fixation, observe the oocytes under a microscope, transfer the cells to a well containing PBST, and wash them three times for five minutes each. PBST is prepared by adding 5 μL of Tween 20 to 10 mL of PBS and mixing thoroughly.

Permeabilization: After washing, transfer the oocytes to a well containing 0.5% Triton X-100 permeabilization buffer and incubate at room temperature in the dark for 30 minutes. Observe the oocytes under a microscope, then transfer the cells to a well containing PBST and wash them three times for five minutes each. The 0.5% Triton X-100 solution is prepared by adding 50 μL of Triton X-100 to 10 mL of PBS and mixing thoroughly.

Blocking: After washing, transfer the oocytes to a well containing 5% BSA blocking solution and incubate at room temperature in the dark for 60 minutes. To prepare the 5% BSA solution, dissolve 0.5 g of BSA powder in 10 mL of PBS, mix thoroughly, and filter the solution using a 0.22 μm syringe filter.

Primary Antibody Incubation: After blocking, transfer the oocytes to a well containing Alpha Tubulin antibody without washing and incubate at room temperature in the dark for 60 minutes. Then, transfer the cells to a well containing PBST and wash them three times for five minutes each. The Alpha Tubulin antibody stock concentration is 1 mg/mL, and it is diluted 1:200 for the experiment by adding 2 μL of the stock to 398 μL of 5% BSA.

Secondary Antibody Incubation: After washing, transfer the oocytes to a well containing Alexa Fluor 488 and incubate at room temperature in the dark for 60 minutes. Then, transfer the cells to a well containing PBST and wash them three times for five minutes each. The Alexa Fluor 488 stock concentration is 2 mg/mL, and it is diluted 1:600 for the experiment by adding 1 μL of the stock to 599 μL of 5% BSA.

DAPI Staining: After washing, transfer the oocytes to a well containing DAPI and incubate at room temperature in the dark for 20 minutes. After staining, transfer the oocytes to a confocal dish containing M2 medium droplets, place them in a humidified chamber, and store them at 4°C until ready for imaging.

#### Cytotoxicity assay of TPA-BT NPs on cumulus cells

Prepare a cumulus cell suspension at an approximate concentration of 5×10^4^ cells/mL. Dispense 100 μL of the suspension into each well of a 96-well plate. Incubate the plate in a CO_2_ incubator (37 °C, 5% CO_2_, with saturated humidity) for 24 hours to facilitate cell adhesion. Establish six experimental groups, each treated with varying concentrations of TPA-BT NPs (0, 20, 50, 100, 250, and 500 μg/mL), ensuring five replicates per group. After adding the NPs, return the plate to the incubator and allow it to incubate for 18 hours. Post-incubation, aspirate the culture medium and wash the cells twice with fresh medium to remove residual NPs, minimizing potential interference. Subsequently, introduce 10 μL of CCK-8 solution to each well and incubate the plate for 1 to 4 hours. Finally, the absorbance at 458 nm was determined using a microplate reader.

#### Live/dead staining assay of cumulus cells exposed to TPA-BT NPs

Prepare a cumulus cell suspension with a concentration of approximately 4×10^5^ cells/mL. Seed the cells into 35 mm cell culture dishes and incubate for 48 hours to allow sufficient cell adhesion to the substrate. Carefully remove the culture medium using a rubber bulb pipette, then wash the dishes twice with PBS, lasting 5 minutes. Prepare the staining solution by combining 10 μL of Calcein-AM and 4 μL of PI with 2 mL of PBS. Add 1 mL of the staining solution to the cells and incubate at 37 °C for 30 minutes. Following incubation, remove the staining solution, replenish it with fresh PBS, and observe the cells under an inverted fluorescence microscope.

#### Effects of TPA-BT NPs on cumulus cell invasiveness

Prepare a cumulus cell suspension with a concentration of approximately 4×10^5^ cells/mL and evenly divide it into four portions, seeding each into a confocal culture dish. Incubate the cells for 48 hours to ensure proper adhesion and expected growth. Subsequently, culture media containing four different concentrations of NPs was introduced to each dish, allowing the cells to co-culture for 18 hours. Following co-culturing, wash the cells three times with PBS to remove any residual NPs. Add 1 mL of 4% paraformaldehyde (PFA) to each dish and incubate for 20 minutes to fix the cells. After fixation, introduce 1 mL of DAPI staining solution to each dish, allowing the cells to stain for 20 minutes. Finally, images of the cells will be captured using confocal laser scanning microscopy (CLSM). Light at a wavelength of 458 nm is utilized as the excitation source to image the TPA-BT NPs within the invasive cells, and the DAPI channel is selected to visualize the chromosomes within the cells.

#### Hemolysis assay

Fresh blood was obtained from female Kunming mice aged 4-6 weeks. Subsequently, the purified red blood cells (RBCs) were isolated from the plasma by dilution with PBS, and the above steps were repeated to clarify the supernatant. The final concentration of 1% (*V*/*V*) RBCs suspension was then mixed with different concentrations of TPA-BT NPs. PBS buffer and ultrapure water were used as negative and positive controls, respectively. Next, after incubation in a water bath at 37 °C for 4 h and centrifugation for 5 min, 100 μL of the supernatant of each sample was transferred to a 96-well plate. The free hemoglobin in the supernatant was measured with a microplate reader at 540 nm. The hemolysis ratio of RBCs was calculated by:

(Equation 1) $$\begin{array}{c} Hemolyticratio\left(\%\right)=\frac{A_{sample}-A_{negative\;control}}{A_{positive\;control}-A_{negative\;control}}\times 100\% \end{array}$$

Where A_sample_, A_negative control,_ and A_positive control_ were denoted as the absorbance of sample, negative and positive control, respectively.

#### TUNEL assay

TUNEL analysis was performed to measure the degree of cellular apoptosis in the ovarian tissue of mice using a TUNEL FITC apoptosis detection kit (Vazyme Biotech Co., Ltd, China) according to the manufacturer’s instructions. The results were obtained under a fluorescent microscope. The nuclei of normal cells were stained blue by DAPI, and the apoptotic cells were labeled green by FITC.

#### Detection of ovarian estrogen receptor

Firstly, the ovarian paraffin sections are processed by deparaffinization and rehydration, and then the sections are blocked with a 5% BSA solution. Incubate the sections with primary antibodies at 37°C for 3 hours (Primary antibodies: Rabbit anti-mouse ERα polyclonal antibody, ab3575, Abcam, USA; Rabbit anti-mouse ERβ polyclonal antibody, ab3576, Abcam, USA). After washing three times with PBS, incubate the sections with the secondary antibody at 37°C for 30 minutes (Secondary antibody: Goat anti-rabbit polyclonal antibody, SV0001, Boster, China). After washing with PBS, perform DAB staining for 3 minutes, where brown staining indicates the expression of the corresponding protein.

#### Detection of serum anti-müllerian hormone and estradiol

Healthy Kunming mice were divided into four groups (6 mice per group), each receiving a different concentration of TPA-BT NPs *via* tail vein injection. After 14 days of continuous maintenance, fresh blood was collected from the mice, and the serum was analyzed for anti-müllerian hormone (AMH) and estradiol using an enzyme-linked immunosorbent assay (ELISA). Set up standard, sample, and blank wells, then proceed with sample addition, washing, and detection solution addition. After stopping the reaction, measure the absorbance at 458 nm using a microplate reader.

#### Counting of preantral follicles

After 14 days of TPA-BT NPs administration, female KM mice (n = 3 per group) were sacrificed, and ovarian tissues were collected. Each ovary was fixed, paraffin-embedded, and sectioned into 10 slices with an interval of 50 μm between consecutive sections. The sections were stained with hematoxylin and eosin (H&E), and the number of preantral follicles was counted under a microscope.

### Quantification and statistical analysis

The relationship between the hormonal content at varying concentrations of mouse injections, ovarian section apoptosis detection, and estrogen receptor detection optical density values was examined using Student’s t-test. P < 0.05 was considered statistically significant. ∗p < 0.05; ∗∗p < 0.01.
