# Supplementary material for: The reproductive toxicity of aggregation-induced emission nanoparticles on mouse ovarian function
Source: iScience. 2025 Dec 2;29(1):114312. doi: 10.1016/j.isci.2025.114312 (PMC12765180; doi:10.1016/j.isci.2025.114312)
Supplement: Document S1. Figures S1–S16 and Tables S1–S6 [file mmc1.pdf]

iScience, Volume *29*

Supplemental information

The reproductive toxicity of aggregation-induced emission nanoparticles on mouse ovarian function

Yibin Zhang, Nan Qiao, Yihang Jiang, Miaozhuang Fan, Wenguang Zhang, Yue Jiao, Zhengzheng Li, Gang Feng, Wing-Cheung Law, Zhourui Xu, and Gaixia Xu

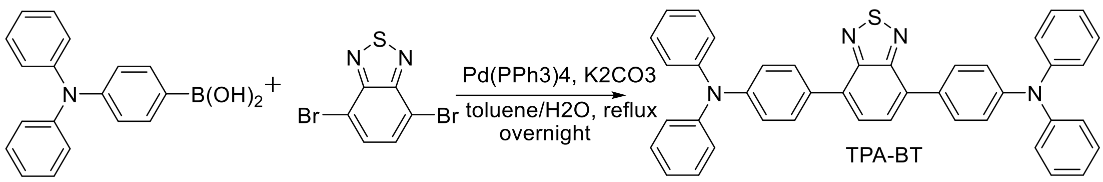

Figure S1. General synthetic procedure for the preparation of TPA-BT.

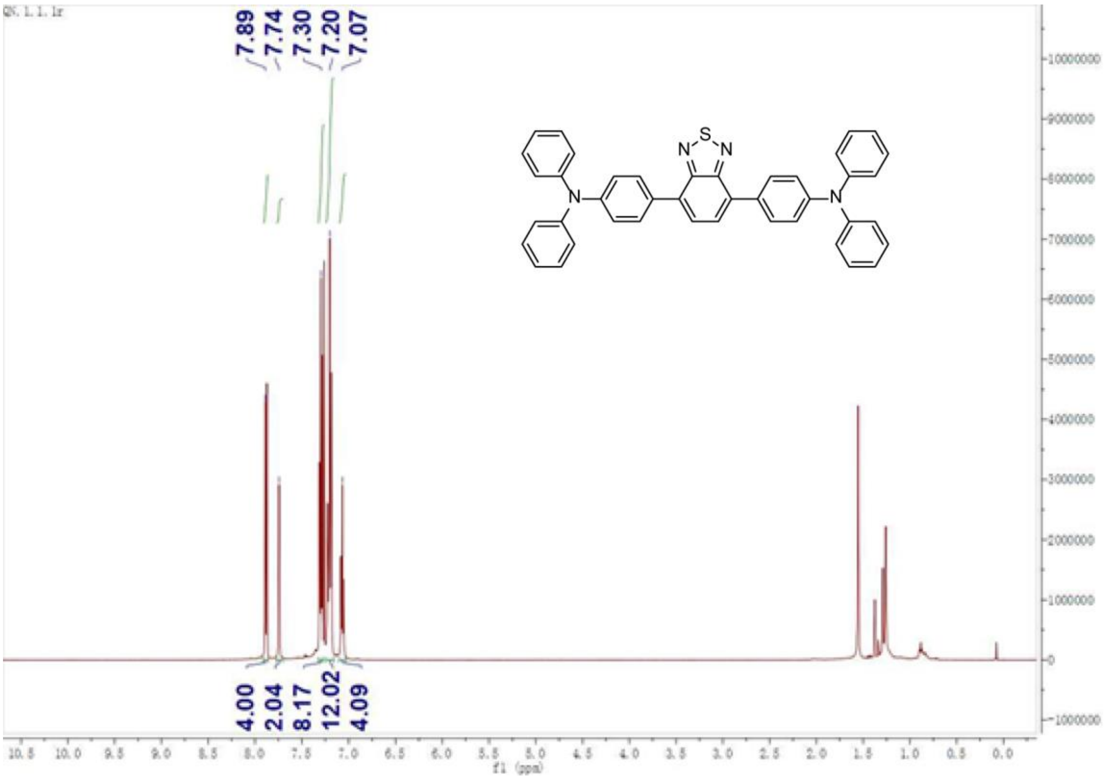

Figure S2. ^{1}H NMR spectrum of TPA-BT.

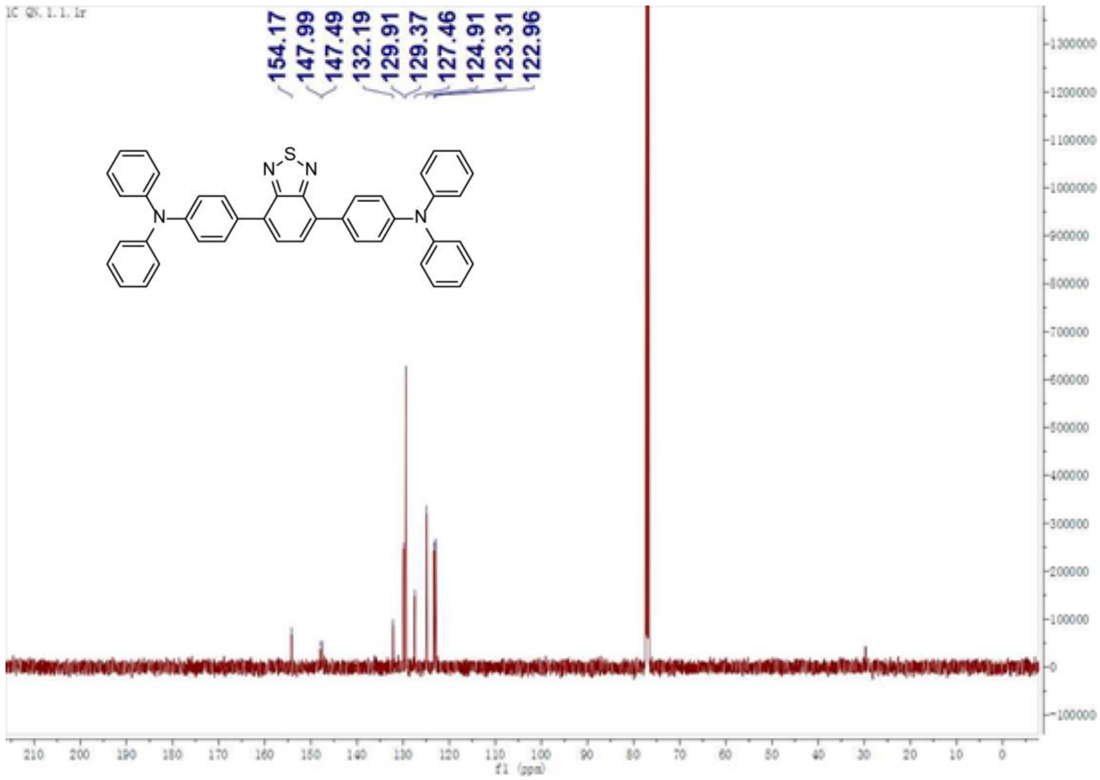

Figure S3. ^{13}C NMR spectrum of TPA-BT.

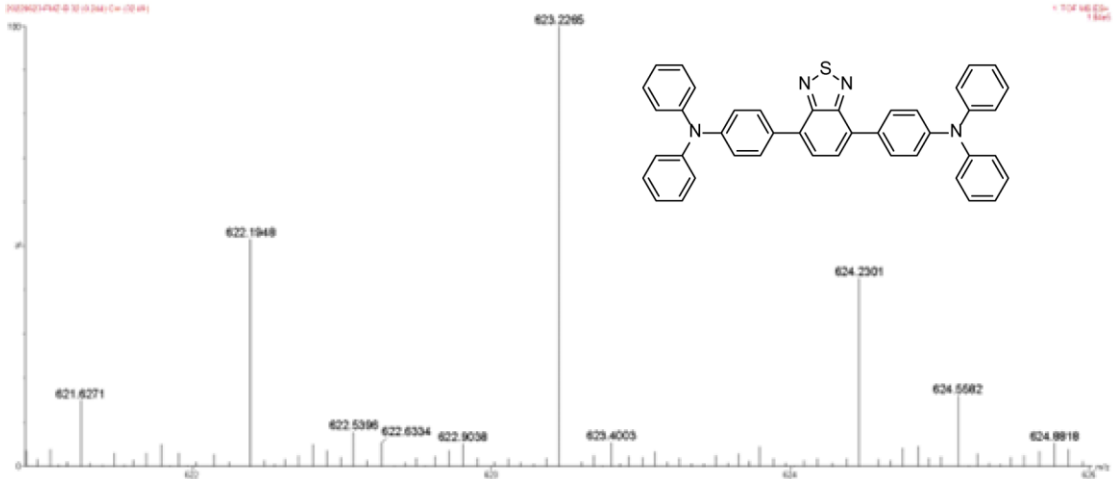

Figure S4. HRMS spectrum of TPA-BT.

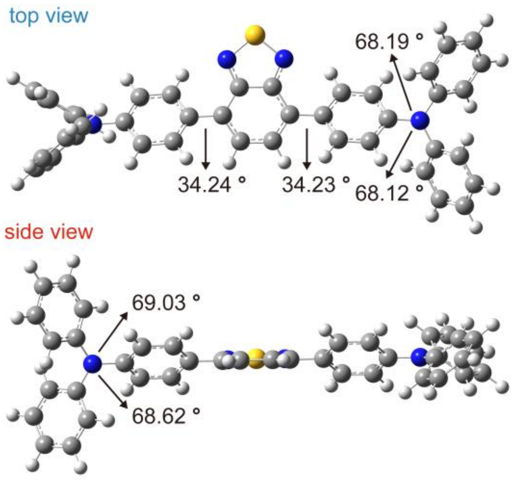

Figure S5. Optimized geometry of TPA-BT and its dihedral angles.

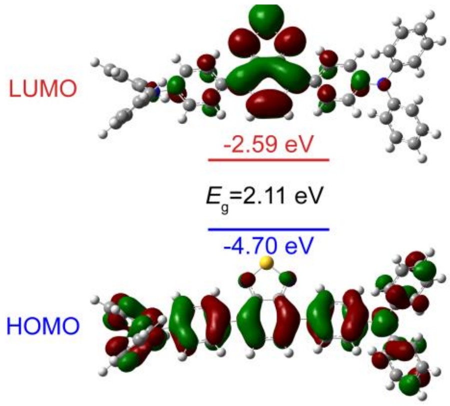

Figure S6. optimized excited-state (S_1) geometries of TPA-BT.

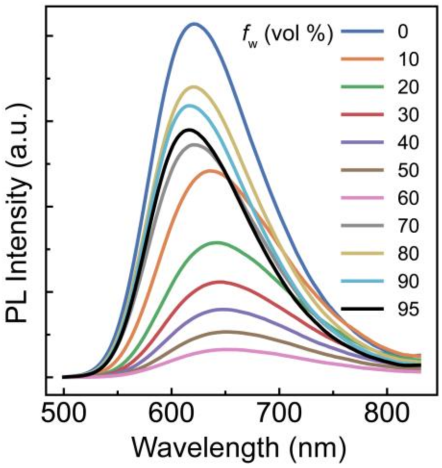

Figure S7. Fluorescence spectra of TPA-BT in THF/water solvent system with different water fractions (f_w).

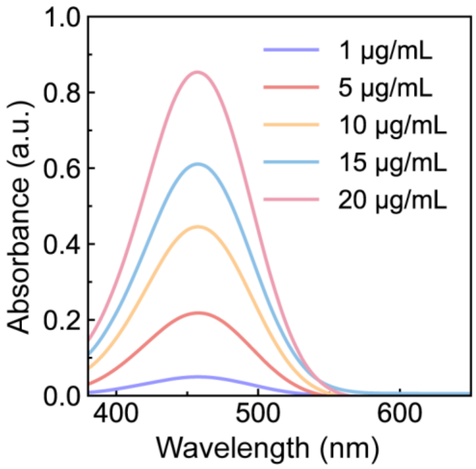

Figure S8. The absorption spectra for various concentrations of TPA-BT.

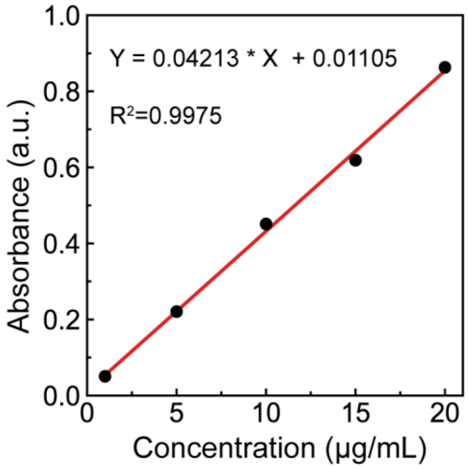

Figure S9. The standard curve of TPA-BT in THF.

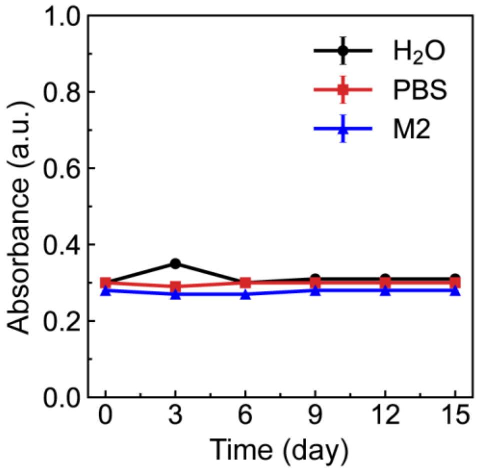

Figure S10. The absorbance of TPA-BT NPs was monitored over 14 days of continuous storage in H_2O, PBS, and M2 medium.

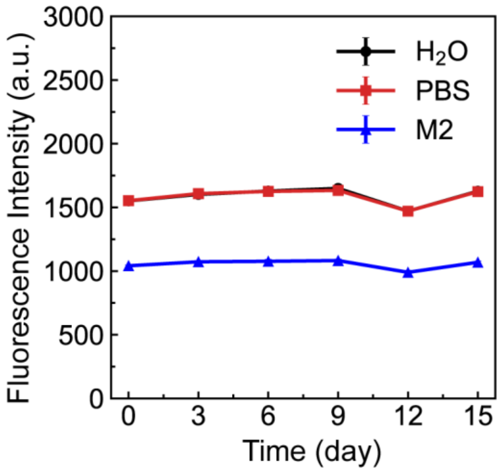

Figure S11. The fluorescence intensity of TPA-BT NPs was monitored over 14 days of continuous storage in H_2O, PBS, and M2 medium.

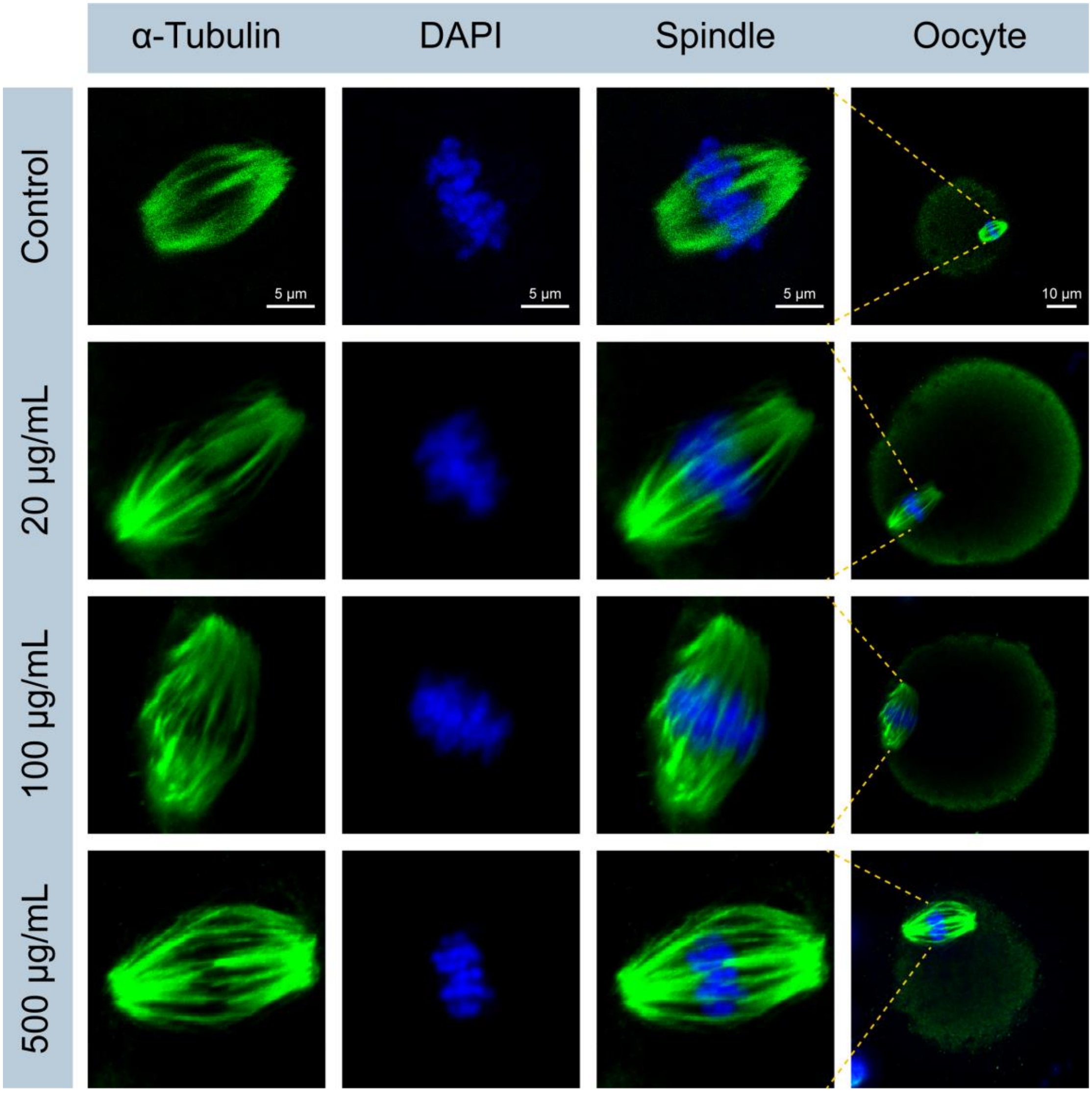

Figure S12. CLSM images of spindle structures of oocytes after 18 hours of *in vitro* culture with varying concentrations of TPA-BT NPs.

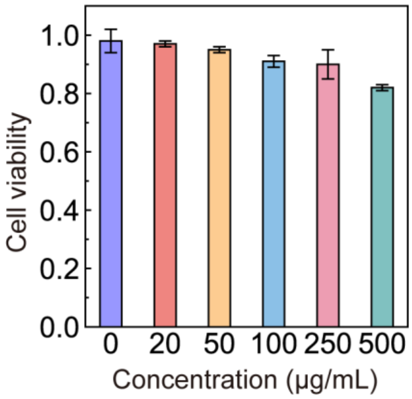

Figure S13. Cell viability of cumulus cells co-culture with TPA-BT NPs.

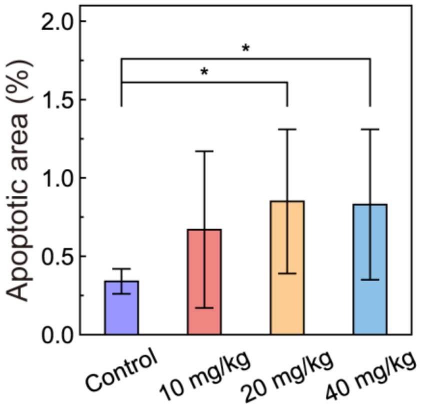

Figure S14. Percentage of ovarian cell apoptosis area.

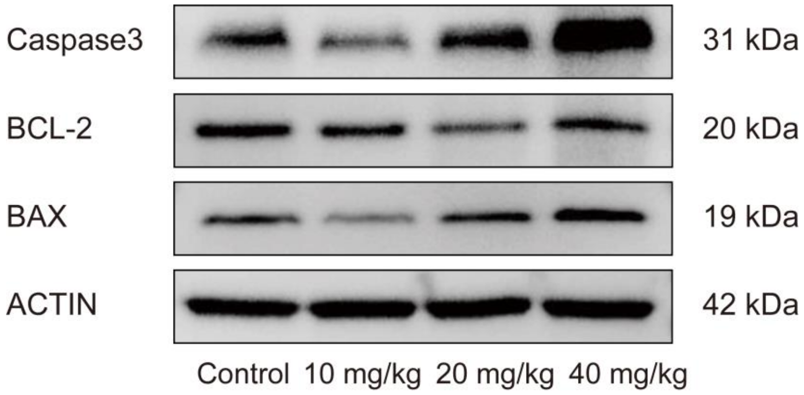

Figure S15. Western blot analysis of apoptosis-related factors in different groups.

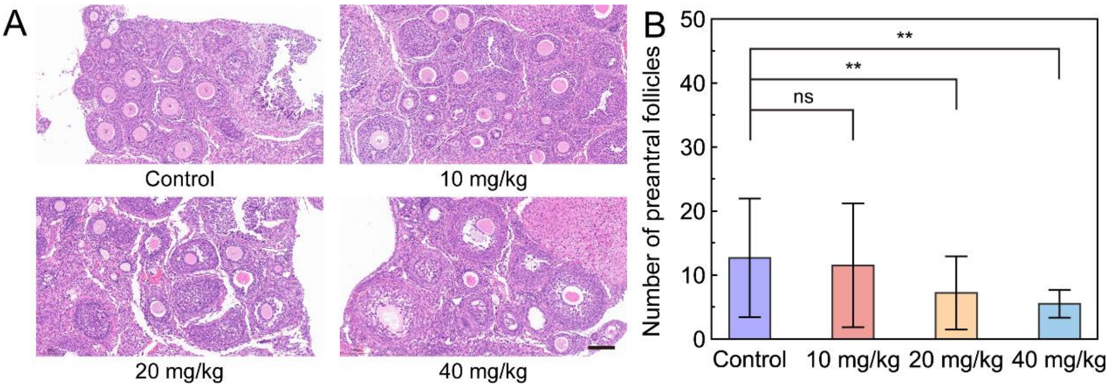

Figure S16. (a) Schematic diagram of the follicular region in HE-stained sections of mouse ovarian tissue. (b) Statistical analysis of the number of preantral follicles in different groups of mice.

Table S1. The HOMO-LUMO distribution and the energy levels and ΔEST values of TPA-BT.

	LUMO [eV]	HOMO [eV]	Bandgap [ΔE_g, eV]	S_1 [eV]	S_2 [eV]	S_3 [eV]	T_1 [eV]	T_2 [eV]	T_3 [eV]	ΔE_{st} [eV]
TPA-BT	-2.27	-4.80	2.53	2.13	2.44	3.32	1.61	2.26	2.61	0.53

Table S2. The number and percentage of oocytes arrested at various stages of meiosis after 18 hours of *in vitro* culture with different concentrations of TPA-BT NPs.

Concentration of TPA-BT NPs	total numbers of cells	GV [number, %]	GVBD [number, %]	PB [number, %]	DG [number, %]
control	202	20, 9.9	36, 17.8	132, 65.3	14, 7.0
20 ppm	205	18, 8.8	51, 24.9	106, 51.7	30, 14.6
100 ppm	214	22, 10.3	36, 16.8	94, 43.9	62, 29.0
500 ppm	228	30, 13.1	62, 27.2	91, 40.0	45, 19.7

Table S3. Blood routine tests for mice treated with PBS buffer and TPA-BT NPs with different concentrations.

Item	Control	10 mg/kg NPs	20 mg/kg NPs	40 mg/kg NPs
WBC (10^9/L)	3.44±0.95	1.94±0.36	4.28±1.93	2.34±0.52
Lymph# (10^9/L)	2.78±0.74	1.52±0.25	3.28±1.66	1.7±0.46
Mon# (10^9/L)	0.12±0.04	0.088±0.02	0.12±0.04	0.1±0.0
Gran# (10^9/L)	0.54±0.22	0.34±0.1	0.88±0.26	0.54±0.19
Lymph (%)	81.22±3.78	78.28±2.86	74.28±4.97	72.16±7.25
Mon (%)	3±0.58	3.66±0.73	3.78±0.68	4±0.97
Gran (%)	15.78±3.25	18.06±2.18	21.94±4.45	23.84±6.31
RBC (10^{12}/L)	9.162±0.7	9.128±0.60	8.644±0.55	9.28±0.24
HGB (g/L)	143±4.77	146.4±9.67	131.4±9.24	141.4±8.21

HCT (%)	47.14±2.57	47.28±3.16	43.26±2.01	45.54±2.45
MCV (fL)	45.6±12.98	51.86±0.89	50.18±1.51	49.12±1.83
MCH (pg)	15.62±0.8	16±0.18	15.16±0.69	15.18±0.58
MCHC (g/L)	303.2±9.15	309±3.74	303±11.51	310±3.16
RDW (%)	15.88±0.55	15.76±0.38	15.4±0.55	16±0.27
PLT (10^9/L)	651.8±156.31	896.8±93.12	840.4±256.62	935.6±202.05
MPV (fL)	5.36±0.55	5.1±0.23	5.46±0.35	5±0.14
PDW (%)	16.9±0.29	16.66±0.27	16.7±0.36	16.3±0.11

The examined parameters include white blood cells (WBC), lymphocyte (Lymph), monocyte (Mon), neutrophile granulocyte (Gran), red blood cells (RBC), hemoglobin (HGB), hematocrit (HCT), mean corpuscular volume (MCV), mean corpuscular hemoglobin (MCH), mean corpuscular hemoglobin concentration (MCHC), red cell volume distribution width (RDW), blood patelet (PLT), mean platelet volume (MPV), platelet distribution width (PDW). The data are shown as mean ± SD (n = 5).

Table S4. Blood biochemistry test for mice treated with PBS buffer and NPs with different concentrations.

Item	Control	10 mg/kg NPs	20 mg/kg NPs	40 mg/kg NPs
ALT (U/L)	44.96±24.04	69.90±25.02	72.30±16.05	56.06±38.26
AST(U/L)	196.98±89.35	191.66±40.26	141.12±27.93	134.64±17.84
ALB (g/L)	44.78±34.94	29.16±6.11	19.54±5.62	22.18±2.64
GLU (mmol/L)	4.02±1.35	4.09±1.19	4.29±1.28	5.12±0.67
TG (mmol/L)	2.13±0.81	1.52±0.3	1.33±0.35	1.34±0.18
UA (umol/L)	112.34±100.78	156.14±82.52	226.84±79.31	213.00±80.91
TC (mmol/L)	3.56±1.68	2.26±0.52	1.94±0.83	2.01±0.29
TBIL (umol/L)	10.16±5.11	9.03±4.51	7.71±3.22	7.59±5.45

The examined parameters include alanine aminotransferase (ALT), aspartate aminotransferase (AST), Albumin (ALB), blood glucose (GLU), triglyceride (TG), uric acid (UA), total cholesterol (TC), and total bilirubin (TBIL). The data are shown as

mean ± SD (n = 5).

Table S5. Zeta potentials of AIEgens NPs.

	TPA-BT NPs (mV)
Day 1	-2.64 ± 0.41
Day 2	-5.56 ± 0.89
Day 3	-3.80 ± 0.76
Day 4	-4.65 ± 1.1
Day 5	-6.12 ± 2.2
Day 6	-9.63 ± 1.4
Day 7	-3.20 ± 1.5

Table S6. The number of preantral follicles in ovarian sections of mice from different groups.

	Control	10 mg/kg	20 mg/kg	40 mg/kg
A1	27	29	19	9
A2	36	38	24	7
A3	35	26	11	6
A4	28	32	16	5
A5	21	24	10	7
A6	20	21	9	4
A7	18	16	6	4
A8	20	8	11	2
A9	17	16	13	5
A10	14	4	11	7
B1	2	8	1	6
B2	9	13	6	3
B3	7	6	4	4
B4	5	6	3	3
B5	8	11	2	4
B6	4	11	2	4
B7	6	8	3	3

B8	6	6	3	4
B9	6	10	2	3
B10	12	5	5	6
C1	8	11	5	9
C2	12	2	2	5
C3	6	2	1	4
C4	13	2	1	8
C5	8	5	3	4
C6	10	4	8	8
C7	4	5	7	7
C8	10	6	5	9
C9	2	1	11	10
C10	6	9	12	5
